# Supplementary material for: Dynamic electron correlations with charge order wavelength along all directions in the copper oxide plane
Source: Nat Commun. 2021 Jan 26;12:597. doi: 10.1038/s41467-020-20824-7 (PMC7838423; doi:10.1038/s41467-020-20824-7)
Supplement: Supplementary file 1 — Supplementary Information [file 41467_2020_20824_MOESM1_ESM.pdf]

Supplementary Information for

Dynamic electron correlations with charge order wavelength along all directions in the copper oxide plane

F. Boschini,[1,2,3] M. Minola,[4] R. Sutarto,[5] E. Schierle,[6] M. Bluschke,[4,6] S. Das,[7] Y. Yang,[7] M. Michiardi,[1,2,8] Y. C. Shao,[9] X. Feng,[9] S. Ono,[10] R. D. Zhong,[11] J. A. Schneeloch,[11] G. D. Gu,[11] E. Weschke,[6] F. He,[5] Y. D. Chuang,[9] B. Keimer,[4] A. Damascelli,[1,2] A. Frano,[7] and E. H. da Silva Neto[12,13,14,*]

[1] *Quantum Matter Institute, University of British Columbia, Vancouver, BC V6T 1Z4, Canada*

[2] *Department of Physics & Astronomy, University of British Columbia, Vancouver, BC V6T 1Z1, Canada*

[3] *Centre Énergie Matériaux Télécommunications, Institut National de la Recherche Scientifique, Varennes, Québec J3X 1S2, Canada*

[4] *Max Planck Institute for Solid State Research, Heisenbergstrasse 1, D-70569 Stuttgart, Germany*

[5] *Canadian Light Source, Saskatoon, Saskatchewan S7N 2V3, Canada*

[6] *Helmholtz-Zentrum Berlin für Materialien und Energie, BESSY II, Albert-Einstein-Str. 15, 12489 Berlin, Germany*

[7] *Department of Physics, University of California San Diego, La Jolla, California 92093, USA*

[8] *Max Planck Institute for Chemical Physics of Solids, Nöthnitzer Straße 40, Dresden 01187, Germany*

[9] *Advanced Light Source, Lawrence Berkeley National Laboratory, Berkeley, CA 94720*

[10] *Central Research Institute of Electric Power Industry, Yokosuka, Kanagawa 240-0196, Japan*

[11] *Condensed Matter Physics and Materials Science, Brookhaven National Laboratory, Upton, NY, USA*

[12] *Department of Physics, University of California, Davis, California 95616, USA*

[13] *Department of Physics, Yale University, New Haven, Connecticut 06511, USA*

[14] *Energy Sciences Institute, Yale University, West Haven, Connecticut 06516, USA*

[*] eduardo.dasilvaneto@yale.edu

Supplementary Note 1: Resonant x-ray scattering

Resonant x-ray scattering provides an enhanced sensitivity to charge modulations for a specific atomic species and orbital. The x-ray beam was tuned resonant to the Cu-L_3 edge (≈ 932 eV) probing charge modulations within the CuO_2 plane [1]. It is reasonable to ask whether the ring-like shape could be related to the core-hole potential in the RIXS experiment. However, we can rule out an interaction between valence electrons and core-holes as the origin of the ring like shape by looking at the energy dependence. Note that the core-hole lifetime can be roughly estimated from the width of the Cu-L_3 resonance (< 0.5 eV). Thus the core-hole lifetime is too short to affect the quasi-static (low-energy) RIXS signal and becomes more influential only at higher energies. Since we do not observe a ring-like feature for the signal above 0.9 eV, we conclude the ring does not originate from the core hole potential.

Supplementary Note 2: Data analysis

EI-RXS: The fluorescence background of the θ-scans displayed in Fig. 3**a** were adjusted using a polynomial function $f(q_x) = A \cdot q_x + B$ in order to match curves for both $q_x < 0.2$ rlu and $q_x > 0.4$ rlu. Background-subtracted curves shown in the lower panel of Fig. 3**a** have been generated by subtracting the high-temperature (300 K) θ-scans. The absolute CO intensity reported in Fig. 3**b** is determined by integrating the area under the background-subtracted CO peaks of Fig. 3**a** normalizing it by the fluorescence background level at q_{CO} for $T = 300$ K. Note that we do not apply any fitting procedure to extract the CO intensity, thus avoiding model-related uncertainty. The doping dependence of q_{CO} in Fig. 3**e** was retrieved by fitting the CO peak position. The cautiously overestimated error bars in Fig. 3**b** represent systematic errors associated with the stability of the instrument (e.g. synchrotron/monochromator stability or thermally induced fluctuations of the sample position) over multiple θ-scans (acquired over several hours), as well as uncertainties associated to the subtraction of the 300 K data.

RIXS: Due to variations of the beam position during a θ scan, the position of the spectrum relative to the spectrometer CCD camera varied slightly over time. To adjust for this, we re-aligned the spectra for each θ and φ to a common reference. 2D-intensity maps shown in Fig. 1**b**–**c** and Fig. 2**a**–**b** of the main text have been generated via a radial (φ) Voronoi interpolation procedure (single traces are shown in Fig. 1**a**). Figure 1**a**–**c** of the main text demonstrates the presence of a 'ring-like' scattering pattern only for the LE signal

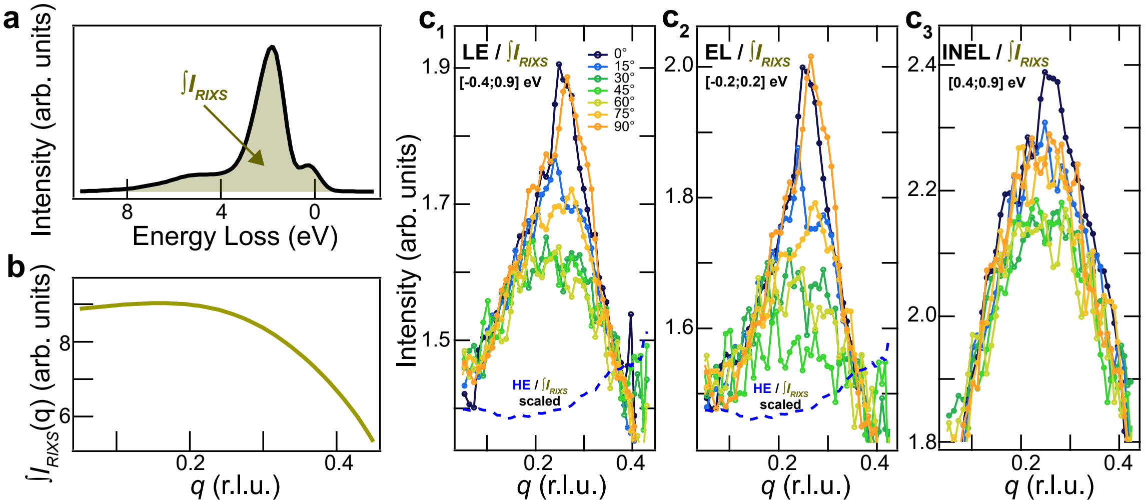

Supplementary Fig. 1. **Normalized RIXS data**. **a** Energy-Loss RIXS spectrum for (q,φ)=(0.05 rlu, 0°). The sepia shadow highlights the total energy-integrated RIXS signal $\int I_{RIXS}$. **b** q-dependence of $\int I_{RIXS}$ for $\varphi = 0°$. **c** Normalized RIXS scattering measured at 50 K as a function of momentum along different φ for different energy ranges: low energy ($\mathbf{c}_1$, LE, $-0.4 < E < 0.9$ eV), elastic ($\mathbf{c}_2$, EL, $-0.2 < E < 0.2$ eV), and inelastic ($\mathbf{c}_3$, INEL, $0.4 < E < 0.9$ eV) regions. The blue dashed line displays the featureless normalized high-energy (HE, $1 < E < 3.5$ eV) signal. Each $(\mathbf{q},\varphi)$ point has been normalized by the related $\int I_{RIXS}(\mathbf{q},\varphi)$.

($E < 0.9$ eV). In an effort to remove any possible geometrical and/or systematic effects, we normalized each $(\mathbf{q},\varphi)$ data point by the corresponding $\int I_{RIXS}(\mathbf{q},\varphi)$ energy-integrated RIXS intensity (see Fig. 1**c**). The sepia shadow in Supplementary Fig. 1**a** highlights the integrated intensity of the RIXS spectrum ($\int I_{RIXS}$). Supplementary Fig. 1**b** displays the q-dependence of the total fluorescence $\int I_{RIXS}$ integrated in the $[-4, 25]$ eV energy range for $\varphi = 0$. Panels **c** show that the φ-dependence of the scattering pattern changes as a function of the selected energy range: LE ($\mathbf{c}_1$, $-0.4 < E < 0.9$ eV), EL ($\mathbf{c}_2$, $-0.2 < E < 0.2$ eV), and INEL ($\mathbf{c}_3$, $0.4 < E < 0.9$ eV), respectively. In agreement with Fig. 1**b**–**c** and Fig. 2**a**–**b** of the main text, the 'ring-like' structure appears only in the INEL component. In addition, note that the normalized HE signal (blue dashed line, $1 < E < 3.5$ eV) is featureless. Following the same approach used for EI-RXS data, normalized RIXS data shown in Supplementary Fig. 1**c** have been marginally adjusted by a linear background to match curves for both $q_x < 0.15$ rlu and $q_x > 0.35$ rlu. The error bars of Fig. 2**c** represent the variation of the normalized scattering intensity at $q = q_{CO}$ for different integration windows (0.1 to 0.19 rlu range).

Supplementary Note 3: Summary of the materials studied

Figure	Material	Doping Level	T_c (K)
3	$Nd_{2-x}Ce_xCuO_4$	0.145	24
3	$Bi_2Sr_{2-x}La_xCuO_{6+\delta}$	0.115	15
3	$Bi_2Sr_2(Ca,Dy)Cu_2O_{8+\delta}$	0.09	37
3	$Bi_2Sr_2(Ca,Dy)Cu_2O_{8+\delta}$	0.097	45
3	$Bi_2Sr_2(Ca,Dy)Cu_2O_{8+\delta}$	0.105	54
1-3	$Bi_2Sr_2CaCu_2O_{8+\delta}$	0.105	54
3	$Bi_2Sr_2CaCu_2O_{8+\delta}$	0.13	75
3	$Bi_2Sr_2CaCu_2O_{8+\delta}$	0.14	82
3	$Bi_2Sr_2CaCu_2O_{8+\delta}$	0.17	91

Supplementary Table 1. **Details of the materials studied for this project.**

Supplementary Note 4: Calculation of the interacting potential

To calculate the form of $V(\mathbf{q})$, we closely follow the model descriptions in Refs. [2, 3]. The interaction term $V(\mathbf{q})$ is the sum of two terms: (i) $U(\mathbf{q})$, the short-term Coulomb repulsion; and (ii) $V_c(\mathbf{q})$, the long-range Coulomb repulsion projected onto the CuO_2 plane. Overall, $V(\mathbf{q}) = U(\mathbf{q}) + V_c(\mathbf{q})$ (see Supplementary Fig. 2). The $\mathbf{q}$-dependence of the short range $U(\mathbf{q})$

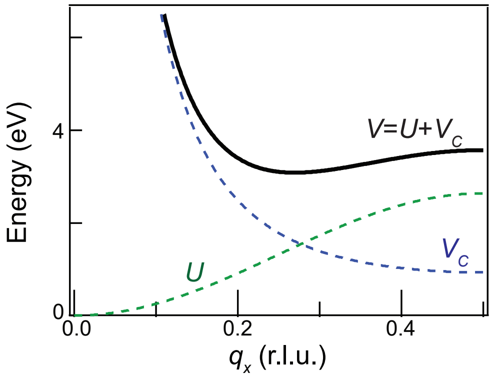

Supplementary Fig. 2. **Contributions to the interaction term $V(\mathbf{q})$.** U (green dashed line) is the short-term Coulomb repulsion, and V_c (blue dashed line) is the long-range Coulomb repulsion projected onto the CuO_2 plane. $V = U + V_c$ is the overall interaction term (black solid line).

can be written up to its next-nearest neighbor as [2, 3]:

$$U(\mathbf{q}) = U_0 + U_1[2 - \cos(q_x) - \cos(q_y)] + U_2[1 - \cos(q_x)\cos(q_y)]. \tag{1}$$

To visualize the in-plane $\mathbf{q}$ structure of $V(\mathbf{q})$ it suffices to calculate U_1 and U_2 [2, 3]:

$$U_1 \propto \frac{t}{2N} \sum_{A,B} \sum_{\mathbf{k}} [\cos(k_x) + \cos(k_y)] f\left(E_{\mathbf{k}}^{A,B}\right); \tag{2}$$

$$U_2 \propto \frac{4t'}{2N} \sum_{A,B} \sum_{\mathbf{k}} [\cos(k_x)\cos(k_y)] f\left(E_{\mathbf{k}}^{A,B}\right), \tag{3}$$

where N is the number of $\mathbf{k}$ points, f is the Fermi-Dirac distribution, and $E_{\mathbf{k}}^{A,B} = \mu - 2t[\cos(k_x) + \cos(k_y)] + 4t'\cos(k_x)\cos(k_y) - 2t''[\cos 2(k_x) + \cos 2(k_y)] \pm t_{\perp}[\cos(k_x) - \cos(k_y)]^2/4$ (A and B are the anti-bonding and bonding bands of Bi2212, respectively). The tight-binding parameters were obtained from recently reported ARPES data [4].

The long range Coulomb component is very general, obtained simply by solving Poisson's equation on a lattice. For a square plane within a 3D tetragonal lattice it follows [5]:

$$V_c(\mathbf{q}) = \frac{v_c}{\sqrt{G_{\mathbf{q}}^2 - 1}}, \quad \text{with} \quad v_c = \frac{ed}{2\epsilon_0\epsilon_{\perp}a^2} \quad \text{and} \quad G_{\mathbf{q}} = 1 + \frac{\epsilon_{||}}{\epsilon_{\perp}}\left(\frac{d}{a}\right)^2 [2 - \cos(q_x) - \cos(q_y)]. \tag{4}$$

Here, e is the electron charge, d the interlayer distance, a the lattice spacing in the CuO_2 plane, ϵ_0 the permittivity of free space, and $\epsilon_{||}$ ($\epsilon_{\perp}$) is the in-plane (out-of-plane) dielectric constant. In addition, the components of the dielectric tensor present in Supplementary Eq. 4 depend on the doping level [6, 7].

The form we use is proportional to q^{-1} at small q. The in-plane Coulomb potential used to describe the MEELS data is $\propto e^{-qd}/q$ [8, 9], which has the same asymptotic behavior towards small q. The layered electron gas model used to described plasmons in electron-doped cuprates [10, 11] has different long-range asymptotic behaviors depending on the value of q_z, varying from approaching a constant ($q_z \neq 0$) to q^{-2} ($q_z = 0, 2\pi$) behavior. In principle, any of the above forms plus the short range behavior could produce a minimum in $V(\mathbf{q})$ in the crossover region from short- to long-range behavior. We note however that the layered electron gas model goes from highly q_z-dependent for small in-plane q to being q_z-independent for large in-plane q. Thus, while q_z has a strong effect for plasmons at low q, it is still unclear what effect it has towards determining the minima of $V(q)$ at the intermediate range. Experimentally, the location of charge order peaks along q_x and q_y are independent of q_z – this is likely also the case for the dynamic scattering ring. Nevertheless, future extensive RIXS investigations will be necessary to resolve the q_z dependence.

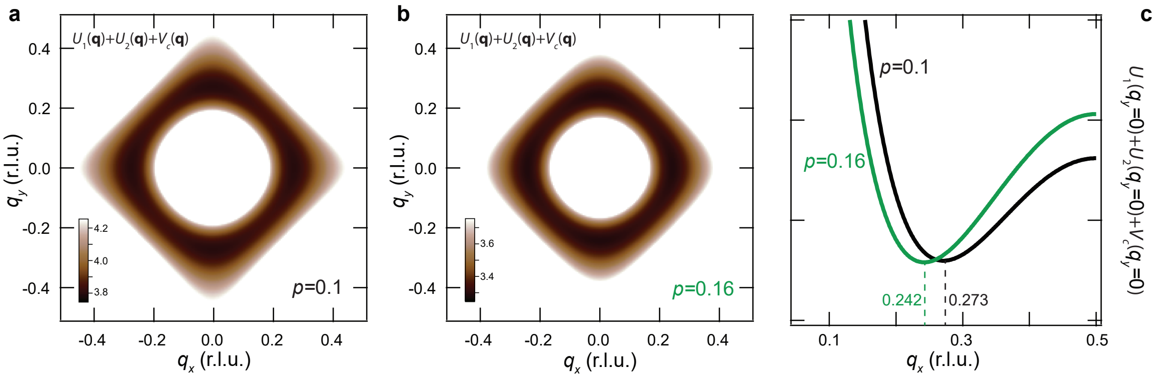

Supplementary Fig. 3. **Doping dependence of q_{CO}. a–b** Momentum structure of the Coulomb repulsion $U_1(\mathbf{q})+U_2(\mathbf{q})+V_c(\mathbf{q})$ for two doping levels of Bi2212 ($a = 3.82$ Å, $d = 15.4$ Å). Parameters for $p = 0.1$: $\epsilon_{||} = 2$, $\epsilon_{\perp} = 10$, $\mu = 0.34\,\mathrm{eV}$, $t = 0.39\,\mathrm{eV}$, $t' = 0.12\,\mathrm{eV}$, $t'' = 0.045\,\mathrm{eV}$, and $t_{\perp} = 0.108\,\mathrm{eV}$. Parameters for $p = 0.16$: $\epsilon_{||} = 2.5$, $\epsilon_{\perp} = 15$, $\mu = 0.37\,\mathrm{eV}$, $t = 0.36\,\mathrm{eV}$, $t' = 0.108\,\mathrm{eV}$, $t'' = 0.036\,\mathrm{eV}$, and $t_{\perp} = 0.108\,\mathrm{eV}$. In an effort to provide a simplified case scenario for the p-dependence of q_{CO} and reduce the number of varying parameters, we assume that the Coulomb repulsion terms (Supplementary Eqs. 2 and 3) do not strongly vary as a function of p. **c** Momentum cuts along (q_x,0) of the Coulomb repulsion shown in **a** ($p = 0.1$, black line) and **b** ($p = 0.16$, green line).

Supplementary Fig. 3**a–b** shows the calculated potential for two doping levels, reproducing the isotropic form found in the experiments. Also note that changes in the band structure and dielectric parameter may also result in small modifications to $V(\mathbf{q})$, which may be responsible for weakly doping dependent q_{CO} (Supplementary Fig. 3**c**). Figures 1**d–e** of the main text were generated using the following parameters: $a = 3.82$ Å, $d = 15.4$ Å, $\epsilon_{||} = 2.5$, $\epsilon_{\perp} = 8$. Regarding the band structure, we used only the antibonding (A) band of Bi2212 with $\mu = 0.274\,\mathrm{eV}$, $t = 0.36\,\mathrm{eV}$, $t' = 0.108\,\mathrm{eV}$, $t'' = 0.036\,\mathrm{eV}$, and $t_{\perp} = 0.108\,\mathrm{eV}$, which correspond to a hole doping $p \approx 0.1$ via a Luttinger count.

Supplementary Note 5: Lindhard approximation

In the framework of the random phase approximation (RPA) the polarizability is $\Pi_{\mathrm{Lind}}(\mathbf{q},\omega)$, which depends on the band structure and Fermi-Dirac distribution function:

$$\Pi_{\mathrm{Lind}}(\mathbf{q},\omega) = \sum_{A,B}\sum_{\mathbf{k},\sigma} \frac{f\left(E^{A,B}_{\mathbf{k+q}}\right) - f\left(E^{A,B}_{\mathbf{k}}\right)}{E^{A,B}_{\mathbf{k+q}} - E^{A,B}_{\mathbf{k}} + \omega}. \tag{5}$$

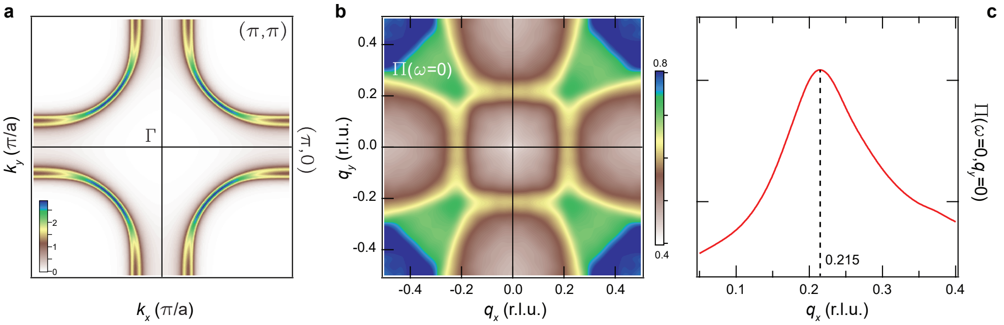

Supplementary Fig. 4. **Fermi surface of Bi2212.** **a** Fermi surface for Bi2212 $p = 0.1$ calculated using tight-binding parameters from Ref. [4] for doping $p = 0.1$ (underdoped 54 K). **b** Momentum dependence of the Lindhard function (Supplementary Eq. 5) calculated using the Fermi surface shown in panel **a**. **c** Lindhard function along the momentum direction (q_x, $q_y = 0$).

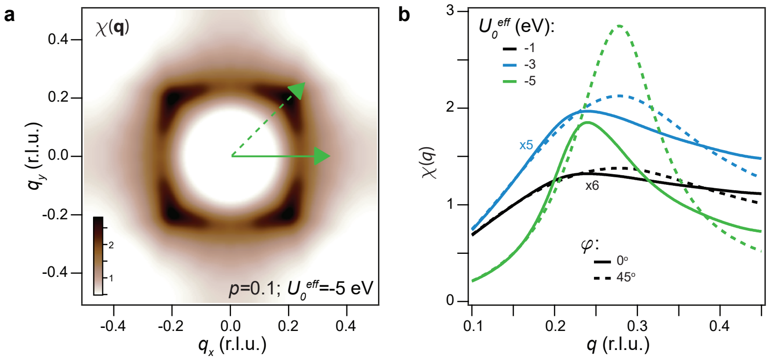

Supplementary Fig. 5. **Anisotropy of $\chi_{\mathbf{RPA}}$.** **a** χ_{RPA} calculated for $p = 0.1$ doping assuming an effective attractive onset $U_0^{\text{eff}} = -5\,\text{eV}$. (**b**) Momentum cuts of χ_{RPA} along $\varphi = 0°$ (solid lines) and $\varphi = 45°$ (dashed lines) as defined in the main text and panel **a**. Different colors indicate different values of U_0^{eff}.

Here the Fermi surface instability mechanism is captured by the static response which is dominated by the Fermi surface, $\Pi_{\text{Lind}}(\mathbf{q}, \omega = 0)$. The doping dependence of q_{CO} has been historically proposed to bear a relation to the change of $E_{\mathbf{k}}^{A,B}$, which would be encoded in the Lindhard function (Supplementary Eq. 5). In particular, the large density of states across the hot-spots at the end of the Fermi arcs could induce the CO instability [12]. However, our Bi2201 data shown in Fig. 3 of the main text demonstrates the presence of a sizable CO even for temperatures higher than the pseudogap onset temperature (T^*), *i.e.* in the absence of hot spots. Supplementary Fig. 4 displays the Fermi surface of underdoped Bi2212 ($p \approx 0.1$, panel **a**, computed using the tight binding parameters described in the Supplementary Note 4), as well as the related Lindhard function and its momentum dependence along

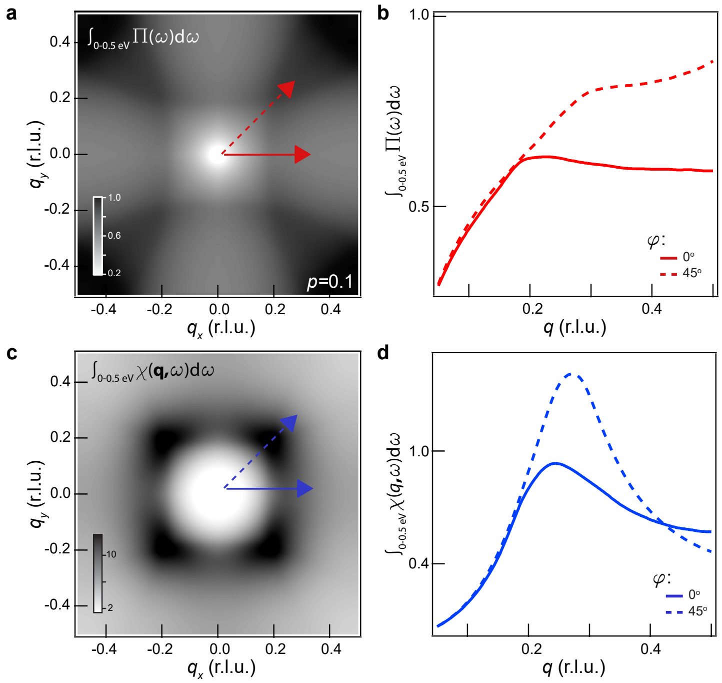

Supplementary Fig. 6. **Energy-integrated $\Pi_{\mathbf{Lind}}$ and $\chi_{\mathbf{RPA}}$.** **a** Momentum dependence of the Lindhard function integrated over MIR energy range ($0 to 0.5\,\mathrm{eV}$). Only the AN band was used for this calculation for $p = 0.1$ doping. **b** Momentum cuts of the energy-integrated Lindhard function along $\varphi = 0°$ (solid lines) and $\varphi = 45°$ (dashed lines) as defined in panel **a**. **c** Momentum dependence of χ_{RPA} integrated over MIR energy range. The Coulomb repulsion of Supplementary Fig. 3**a** was used. **d** Momentum cuts of χ_{RPA} along $\varphi = 0°$ (solid lines) and $\varphi = 45°$ (dashed lines) as defined in panel **c**.

(q_x,0) in panels **b** and **c**, respectively. Note that the maximum of the Lindhard function, Supplementary Eq. 5, calculated for the normal state Fermi surface (*i.e.* for $T > T^*$), falls short of the experimental q_{CO} (see Supplementary Fig. 4**c**). *Regardless, the most relevant qualitative feature of the Lindhard function to our studies is that it displays a C_4 symmetry, with square-like contours, in contrast to the circular symmetry (C_∞) reported in Figs. 1 and 2 of the main text.*

To demonstrate the square-like structure of the static RPA susceptibility, we perform a simple proof-of-principle calculation using the following formula:

$$\chi_{\mathrm{RPA}}(\mathbf{q}, \omega = 0) = \frac{\Pi_{\mathrm{Lind}}(\mathbf{q}, \omega = 0)}{1 + V(\mathbf{q})\Pi_{\mathrm{Lind}}(\mathbf{q}, \omega = 0)}. \quad (6)$$

The results show deviations from the isotropic form observed in our experiments. This is well displayed in Supplementary Fig. 5**a** where χ_{RPA} is calculated using the Lindhard function of Supplementary Fig. 4**b**, the Coulomb repulsion of Supplementary Fig. 3**a**, and assuming

an effective attractive onset $U_0^{\text{eff}} = -5\,\text{eV}$. To show the weak dependence of the anisotropy of χ_{RPA} on the value of U_0^{eff}, we display momentum cuts along (q_x,0), solid lines, and the diagonal, dashed lines, for three different values of U_0^{eff}, Supplementary Fig. 5**b**.

We showed that the Lindhard function and RPA susceptibility deviate significantly from a circular scattering pattern in the $\omega = 0$ static limit (see Supplementary Figs. 4–5). However, the momentum-pattern of both $\Pi_{\text{Lind}}(\mathbf{q}, \omega)$ and $\chi_{\text{RPA}}(\mathbf{q}, \omega)$ may change when integrated over ω. Supplementary Fig. 6**a–b** displays Π_{Lind} integrated over the MIR energy range (0 to 0.5 eV). The energy-integrated Π_{Lind} deviates even more from a a circular pattern, making Π_{Lind} almost featureless as a function of the momentum q (see Supplementary Fig. 6**b**). However, the energy-integrated χ_{RPA} *approaches* a circular pattern in q_x-q_y (Supplementary Fig. 6**c–d**). This happens because the $\mathbf{q}$ space patterns in $\Pi_{\text{Lind}}(\mathbf{q}, \omega)$ disperse and broaden with energy, which causes them to be washed out by the energy summation. At the same time, $V(\mathbf{q})$ is energy independent, allowing it to more strongly influence χ_{RPA}, relative to the contribution from Π_{Lind}.

---

[1] R. Comin and A. Damascelli, Annual Review of Condensed Matter Physics **7**, 369 (2016).

[2] G. Seibold, F. Becca, F. Bucci, C. Castellani, C. Di Castro, and M. Grilli, The European Physical Journal B - Condensed Matter and Complex Systems **13**, 87 (2000).

[3] S. Caprara, C. Di Castro, G. Seibold, and M. Grilli, Phys. Rev. B **95**, 224511 (2017).

[4] I. K. Drozdov, I. Pletikosić, C.-K. Kim, K. Fujita, G. D. Gu, J. C. S. Davis, P. D. Johnson, I. Božović, and T. Valla, Nature Communications **9**, 5210 (2018).

[5] F. Becca, M. Tarquini, M. Grilli, and C. Di Castro, Phys. Rev. B **54**, 12443 (1996).

[6] J. Hwang, T. Timusk, and G. D. Gu, Journal of Physics: Condensed Matter **19**, 125208 (2007).

[7] T. Takayanagi, M. Kogure, and I. Terasaki, Journal of Physics: Condensed Matter **14**, 1361 (2002).

[8] M. Mitrano, A. A. Husain, S. Vig, A. Kogar, M. S. Rak, S. I. Rubeck, J. Schmalian, B. Uchoa, J. Schneeloch, R. Zhong, G. D. Gu, and P. Abbamonte, Proceedings of the National Academy of Sciences **115**, 5392 (2018).

[9] A. A. Husain, M. Mitrano, M. S. Rak, S. Rubeck, B. Uchoa, K. March, C. Dwyer, J. Schneeloch, R. Zhong, G. D. Gu, and P. Abbamonte, Phys. Rev. X **9**, 041062 (2019).

[10] M. Turlakov and A. J. Leggett, Phys. Rev. B **67**, 094517 (2003).

[11] M. Hepting, L. Chaix, E. W. Huang, R. Fumagalli, Y. Y. Peng, B. Moritz, K. Kummer, N. B.

Brookes, W. C. Lee, M. Hashimoto, T. Sarkar, J.-F. He, C. R. Rotundu, Y. S. Lee, R. L. Greene, L. Braicovich, G. Ghiringhelli, Z.-X. Shen, T. P. Devereaux, and W. S. Lee, Nature **563**, 374 (2018).

[12] R. Comin, A. Frano, M. M. Yee, Y. Yoshida, H. Eisaki, E. Schierle, E. Weschke, R. Sutarto, F. He, A. Soumyanarayanan, Y. He, M. Le Tacon, I. S. Elfimov, J. E. Hoffman, G. A. Sawatzky, B. Keimer, and A. Damascelli, Science **343**, 390 (2014).
